# The Complement Receptors C3aR and C5aR Are a New Class of Immune Checkpoint Receptor in Cancer Immunotherapy

**DOI:** 10.3389/fimmu.2019.01574

**Published:** 2019-07-19

**Authors:** Yu Wang, Hui Zhang, You-Wen He

**Affiliations:** ^1^Life Science Institute, Jinzhou Medical University, Jinzhou, China; ^2^First Affiliated Hospital, China Medical University, Shenyang, China; ^3^Department of Immunology, Duke University Medical Center, Durham, NC, United States

**Keywords:** complement, cancer immuno therapy, complement receptor C3aR, complement receptor C5aR, IL-10 (interleukin-10), PD-1 - PDL-1 axis, immune check point

## Abstract

Cancer immunotherapy has made remarkable clinical advances in recent years. Antibodies targeting the immune checkpoint receptors PD-1 and CTLA-4 and adoptive cell therapy (ACT) based on *ex vivo* expanded peripheral CTLs, tumor infiltrating lymphocytes (TILs), gene-engineered TCR- and chimeric antigen receptor (CAR)-T cells have all shown durable clinical efficacies in multiple types of cancers. However, these immunotherapeutic approaches only benefit a small fraction of cancer patients as various immune resistance mechanisms and limitations make their effective use a challenge in the majority of cancer patients. For example, adaptive resistance to therapeutic PD-1 blockade is associated with an upregulation of some additional immune checkpoint receptors. The efficacy of transferred tumor-specific T cells under the current clinical ACT protocol is often limited by their inefficient engraftment, poor persistence, and weak capability to attack tumor cells. Recent studies demonstrate that the complement receptor C3aR and C5aR function as a new class of immune checkpoint receptors. Complement signaling through C3aR and C5aR expressed on effector T lymphocytes prevent the production of the cytokine interleukin-10 (IL-10). Removing C3aR/C5aR-mediated transcriptional suppression of IL-10 expression results in endogenous IL-10 production by antitumor effector T cells, which drives T cell expansion and enhances T cell-mediated antitumor immunity. Importantly, preclinical, and clinical data suggest that a signaling axis consisting of complement/C3aR/C5aR/IL-10 critically regulates T cell mediated antitumor immunity and manipulation of the pathway *ex vivo* and *in vivo* is an effective strategy for cancer immunotherapy. Furthermore, a combination of treatment strategies targeting the complement/C3aR/C5aR/IL-10 pathway with other treatment modalities may improve cancer therapeutic efficacy.

## Introduction

As a major component of the innate immunity, the complement system also directly regulates lymphocyte function (1, 2). Recent studies have shed important insights to the role of complement and its receptors in antitumor immunity. Clinical observations and animal studies suggest that complement signaling inhibits antitumor immunity. It was reported that tumor or circulating complement levels are positively correlated with tumor size and poor outcome in different types of cancers, such as neuroblastoma, colorectal, lung, ovarian cancer, chronic lymphocytic leukemia, and carcinomas of the digestive tract (3). Extensive animal studies have also demonstrated that the complement system functions to inhibit antitumor immunity (4–15). Mechanistically, complement may inhibit antitumor immunity by promoting recruitment of myeloid-derived suppressor cells (MDSCs) into the tumor microenvironment (TME) (4–6, 9, 12, 13) or by suppressing dendritic cells (DCs)/NK cell activation (7, 8). Recent studies suggest that a new mechanism plays an important role in complement signaling-mediated suppression of antitumor immunity: direct inhibition of IL-10 production in CD8^+^ tumor infiltrating lymphocytes (TILs) in TME (11, 14). Here, we summarize relevant findings and propose that C3aR and C5aR function as a new class of immune checkpoint receptors that should be targeted for cancer immunotherapy.

## Complement Suppresses Antitumor Immunity Through C3aR and C5aR

In addition to the many clinical reports positively correlating complement levels with tumor size and poor outcome in various human cancers [reviewed by Pio et al. (3)], animal studies testing different tumor types in different mouse models also show that the complement signaling pathway exerts potent inhibition on antitumor immunity (4–15) (Table 1). The tested mouse tumor models include TC-1 cervical cancer, Lewis lung cancer, RMA lymphoma, 4T1, and E0771 breast cancer, B16 melanoma, HPV16 skin cancer, and MC38 colon cancer with either transplanted tumor cells or spontaneously developed cancers. Complement signaling was disrupted in these animal studies by using genetic models including mice deficient for C3, C4, C3aR, or C5aR1 (4, 9–15) or inhibitors to complement C3, C3aR, and C5aR1 (4–6, 8, 10–12, 14). The reported results are highly consistent in that tumor growth is suppressed when complement-mediated signaling is inhibited or removed. In studying the underlying cellular mechanisms, Markiewski and colleagues first showed that C5a/C5aR1 interaction promotes the migration of MDSCs into tumors and enhances the suppressive capacity of tumor-associated MDSCs (4). The regulation of myeloid suppressor cells in tumors by complement signaling is also observed by several other studies (5, 6, 9, 12, 13). Thus, a major immune suppressive role by complement signaling may be mediated through recruitment of MDSCs into tumors. In addition to MDSCs, other innate cell populations such as neutrophils, DCs and NK cells are also involved in complement-mediated immune suppression of antitumor immunity (7, 8, 10). When complement C3 is exhausted using cobra venom factor, NK cells are greatly increased in tumors and depletion of NK cells nullifies the enhanced antitumor activity induced by cobra venom factor treatment (8). Although complement signaling modulates innate immune cell activities, the enhanced antitumor immunity exhibited in mice following disruption of complement signaling is T lymphocyte dependent. Not only effector CD4^+^ and CD8^+^ TILs are enhanced in these mice but also depletion of T cells through TCRα genetic deletion or antibodies against CD4^+^ or CD8^+^ T cells diminishes the enhanced antitumor immunity in the complement signaling deficient models (4, 8–15). These studies suggest that multiple immune suppressive mechanisms are induced by C3aR and C5aR1 signaling (Table 1).

**Table 1:** Mouse models on the complement/C3aR/C5aR1/IL-10 pathway in antitumor immunity.

**Reference**	**Animal strain/Treatment**	**Tumor type**	**Phenotype**
Ajona et al. (12)	PD-1/C5a double blockade	Lung cancer	Growth and metastasis inhibition
Cho et al. (16)	C3^−/−^ mice, C5a silencing in tumor	Ovarian cancer	C5a recruits MDSCs to tumor microenvironment
Corrales et al. (5)	C5aR antagonist	Lung cancer	C5a recruits MDSCs to tumor microenvironment
Emmerich et al. (17)	IL10Rb^−/−^ mice, IL-10 treatment	Squamous carcinoma	IL-10 promotes anti-tumor CD8^+^ T cell response
Gunn et al. (6)	SCID mice, C5a overexpression	Lymphoma/ovarian cancer	C5a recruits MDSCs
Janelle et al. (8)	cobra venom factor treatment	Melanoma	Complement inhibits NK function
Kwak et al. (14)	C3^−/−^ mice,C3aR, C5aR antagonists	lung Cancer	Complement inhibits CD4^+^ T cell function
Markiewski et al. (4)	C3^−/−^, C4^−/−^, factor B^−/−^, C5aR^−/−^ mice	Cervical cancer	Complement recruits MDSCs to tumor
Medler et al. (15)	K14-HPV16 Tg, C3^−/−^mice	Squamous cell carcinoma	C5a/C5aR regulate macrophage/mast cell
Mumm et al. (18)	IL-10^−/−^, IFNg^−/−^, MMTV-rtHer2 Tg mice	Squamous tumor/thymoma	IL-10 promotes CD8^+^ T cell function
Nabizadeh et al. (10)	C3aR^−/−^mice, C3aR/C5aR antagonists	melanoma, colon, breast cancer	Complement inhibits CD4^+^ T cell and neutrophil
Qing et al. (7)	C3^−/−^ and C5aR^−/−^ mice	Melanoma	Complement inhibits DC-NK function through MDSCs
Vadrevu et al. (9)	C5aR^−/−^ mice, C5aR antagonist	Breast cancer	Complement inhibits T cell through Treg and MDSCs
Wang et al. (11)	C3^−/−^, IL-10^−/−^, TCR^−/−^mice, C3aR and C5aR antagonists	Melanoma/colon/breast cancer	Complement inhibits antitumor CD8^+^ T cell by
Zha et al. (13)	C5aR^−/−^ mice, PD-1 blockade and C5aR antagonist	Melanoma/colon cancer	C5a/PD-1 blockade enhances antitumor efficacy

## C3aR and C5aR-Mediated Immune Suppression on T Lymphocytes

Three small cationic peptides, C3a, C4a, and C5a, generated by complement activation are termed as anaphylatoxins. These peptides induce chemotaxis, cell activation, and inflammatory signaling by binding to their respective G-protein-coupled receptors (GPCR), referred to as C3aR and C5aR1. The models for anaphylatoxins binding to their cognitive receptors have been proposed after the molecular cloning of C3aR and C5aR1 (19). In the immune system, C3aR is predominantly distributed on leukocytes of myeloid lineages, such as neutrophils, basophils, eosinophils, mast cells, monocytes/macrophages (20–23). Ligand-receptor engagement induced receptor phosphorylation leads to receptor desensitization, internalization, and activation of diverse downstream signaling pathways in different cell types. C3aR is highly expressed on neutrophils, and C3a induces calcium influx in response to C3a (24); however, C3aR inhibits neutrophil mobilization *in vivo* in an intestinal ischemia-reperfusion model (25). In mast cells, C3a activates PI3K signaling pathways and subsequent Akt-phosphorylation, as well as MAP kinases Erk1/Erk2 to promote cytokine expression (26). In human monocyte/macrophage, engagement of C3a to Ca3R, together with TLR signaling induces secretion of proinflammatory cytokines such as IL-1β, IL-6, and TNFα (27, 28). C3aR signaling modulates IL-1β secretion through NLRP3 inflammasome activation by regulating ATP efflux (29). Similar to C3aR, C5aR is abundantly expressed in neutrophils, eosinophils and basophils, monocytes/macrophages, and mast cells (30–33). C5a binding to C5aR causes calcium flux as well as activation of several components of different signaling pathways, including PI3K-γ kinase, phospholipase C, phospholipase D and Raf-1/B-Raf mediated activation of MEK-1 (34–37). In addition to a similar proinflammatory function of C3aR, C5aR1 is also a chemotactic receptor. Upon engagement with C5aR1, C5a serves as a chemoattractant for monocytes, neutrophils, eosinophils, and basophils (38).

It is well established that complement components and their receptors C3aR and C5aR1 are expressed in not only myeloid and tumor cells but also CD4^+^ T lymphocytes (39–44). Furthermore, endogenously or locally produced C3a and C5a bind to C3aR and C5aR on CD4^+^ T cells and regulate T cell function, such as differentiation, survival and cytokine production (40, 41, 45, 46). Interestingly, in contrast to the lack of C3aR and C5aR1 expression on peripheral CD8^+^ T cells in naive mice, both receptors are strongly upregulated on CD8^+^ TILs from mouse and human tumors (11). Overall, ~20% of CD8^+^ TILs are C3aR and C5aR double positive. To determine the source of complement that mediates immune suppression on CD8^+^ TILs, chimeric mice with either lymphocytes or host cells lacking C3 were used as tumor-bearing hosts. C3-deletion in CD8^+^ T cells was sufficient to remove complement-mediated suppression on antitumor immunity (11), suggesting that autocrine C3 production and the interaction of activation products with Ca3R/C5aR plays a critical role in suppressing effector CD8^+^ TIL function.

## C3aR and C5aR Signaling Inhibits IL-10 Production in Tumor Infiltrating T Lymphocytes

How does autocrine complement signaling inhibit effector CD8^+^ T cell function? Several clues suggest a possible mechanism underlying C3aR/C5aR signaling-mediated immune checkpoint function: complement signaling may inhibit IL-10 production in effector T lymphocytes given the role of IL-10 in CD8^+^ TIL expansion and immune activating function in antitumor immunity (see Discussion in next section). First, it was shown that a fraction of CD8^+^ effectors expresses IL-10 at the peak of coronavirus infection and the IL-10^+^CD8^+^ T cells show superior CTL activity and *in vivo* protection against chronic infection (47). Second, we found that complement pathway related genes are enriched in the IL-10^+^CD8^+^ T cells (11), suggesting a possibility of mutual or reciprocal regulation. Indeed, in C3^−/−^ *Il10* reporter (Tiger) mice, CD8^+^ TILs within B16 tumors but not peripheral blood readily express IL-10 (11). Kwak and colleagues also observed enhanced IL-10 expression in CD4^+^ and CD8^+^ T lymphocytes in lungs of tumor-bearing C3-deficient mice (14). Furthermore, antagonists to C3aR and C5aR1 also promote IL-10 production in CD8^+^ TILs as well as *in vitro* activated CD8^+^ T cells (11). Importantly, the enhanced antitumor immunity in complement-deficient mice or wildtype mice treated with antagonists to C3aR and C5aR1 depend on IL-10. Depletion of the IL-10 gene in these mice completely abolishes the enhanced antitumor immunity in both melanoma and breast cancer tumor-bearing C3-deficient mice (11). The suppression of IL-10 production in CD8^+^ TILs is mediated through endogenously produced complement and its autocrine interaction with C3aR and C5aR on CD8^+^ T cells. The inhibition on IL-10 production by signaling through C3aR and C5aR is redundant as antagonism to one of these receptors alone does not promote IL-10 production. Accordingly, antagonism to C3aR and C5aR1, but not to a single receptor, suppresses tumor growth and the antitumor effect depends on IL-10 *in vivo* (11). Therefore, inhibition of antitumor immunity through suppression of IL-10 production in CD8^+^ TILs in response to complement/C3aR/C5aR signaling represents a new mechanism of complement-mediated immune supression (48).

## IL-10 Functions as an Immune Activating Cytokine in Cancer Immunotherapy

IL-10 is a pleiotropic cytokine produced by many cell populations, including but not limited to activated T cells, B cells, macrophages as well as mast cells (49, 50). Although it was initially identified as a cofactor for thymocytes growth and T cell activation, IL-10 was further recognized as a regulatory cytokine due to its anti-inflammatory functions. IL-10 impairs the maturation of dendritic cells and macrophages by interfering with upregulation of costimulatory molecules such as CD80, CD86, MHCII, and CD83 on activated dendritic cells and macrophages (51, 52). In addition, IL-10 skews the Th1/Th2 balance to Th2 by selectively blocking IL-12 synthesis in activated dendritic cells (53). Macrophages can be polarized to M1 (inflammatory) or M2 (anti-inflammatory) phenotypes depending on the microenvironmental stimuli. IL-10 inhibits the activation and proliferation through Stat3-dependent and -independent pathways and polarizes macrophage to a M2 like phenotype (54, 55). IL-10 directly acts on CD4^+^ T cells to differentiate T helper cells into inducible regulatory T cells and maintain the expression of key transcription factor Foxp3 (56, 57). Regulatory T cells also express IL-10 and mice deficient for IL-10 in regulatory T cells did not display systemic autoimmunity; however, these mice developed spontaneous colitis, skin and lung hyperreactivity, suggesting an organ specific role of IL-10 on regulatory T cells (58).

Although IL-10 is often associated with an immune suppressive function, recent clinical studies have unequivocally shown that IL-10 is an immune activating cytokine promoting antitumor immunity (59–61). In a phase I clinical trial, pegylated recombinant human IL-10 (rhIL-10) has shown encouraging clinical efficacy in several types of solid tumors (59). Among the 24 patients treated with rhIL-10 monotherapy at 20–40 μg/kg active dose, the overall objective response rate is 21%. Furthermore, IL-10 treatment increases serum levels of pro-inflammatory cytokines IL-18 and IFNγ as well as FasL in cancer patients and the induced cytokine levels are strongly correlated with clinical responses (59, 61). Pegylated rhIL-10 treatment dramatically expands PD-1^+^LAG-3^+^ activated CD8^+^ T cells in the blood of cancer patients. Importantly, both the number and effector function of CD8^+^ TILs from these patients are increased (61). These results support that the major function of rhIL-10 is to expand the number as well as enhance the effector function of antitumor CD8^+^ T cells in cancer patients. A Phase 3 clinical trial (NCT02923921) in patients with metastatic pancreatic cancer is being conducted based on promising efficacy data from early clinical studies. Mechanistically, checkpoint inhibition, and IL-10 treatment together enhances the number and quality of pre-existing TILs. The efficacy of PD-1/PD-L1 inhibitors is highly associated with tumor microenvironment such as TIL density, PD-1/PD-L1 expression; tumor intrinsic feature, such as tumor mutational burden, microsatellite instability; as well as gut microbiota (62). The clinical trials of pegylated recombinant human IL-10 are focused on several solid tumor types. Its efficacy on solid and blood tumor types needs to be tested clinically in the future.

Another potential application for IL-10 and C3aR/C5aR antagonists is to incorporate them into *in vitro* expansion protocols of T cells for ACT. IL-2 is the primary cytokine used in the *in vitro* expansion of TILs and gene-engineered T cells for clinical use, however, the *in vivo* efficacy of expanded T cells under the current clinical ACT protocol is often limited by their inefficient engraftment, poor persistence, and weak capability to attack tumor cells (63–68). It was shown long ago that IL-10 augments IL-2-induced proliferation and promotes CTL activity of activated CD8^+^ T cells (69–72). Consistent with animal studies and human clinical trial data showing that IL-10 promotes CD8^+^ TIL proliferation (11, 17, 18, 61), addition of IL-10 to *in vitro* culture of TILs from human lung cancer with IL-2 drastically enhances the quantity and quality of the expanded human TILs and upregulates genes related to several signaling pathways, such TCR signaling, Notch signaling, cell cycle and CTL killing (11). Furthermore, pegylated rhIL-10 also prevents continuous TCR-stimulation induced apoptosis of activated human T cells (61). Interestingly, remissions in lymphoma patients treated with anti-CD19 chimeric antigen receptor (CAR-T) cells are associated with high serum levels of IL-10 and IL-15 (73). These results strongly suggest that the addition of IL-10 to the IL-2-supported *in vitro* T cell expansion protocol may improve the clinical efficacy of adoptive T cell therapy. In addition to IL-10, antagonists to C3aR/C5aR1 may also be used in such protocol as the *in vitro* culture of activated CD8^+^ T cells in the presence of C3aR/C5aR1 antagonists induces IL-10 production (11).

## Synergistic Effect by Targeting Complement/C3aR/C5aR/IL-10 Pathway and Other Treatment Modalities

**Figure 1:**
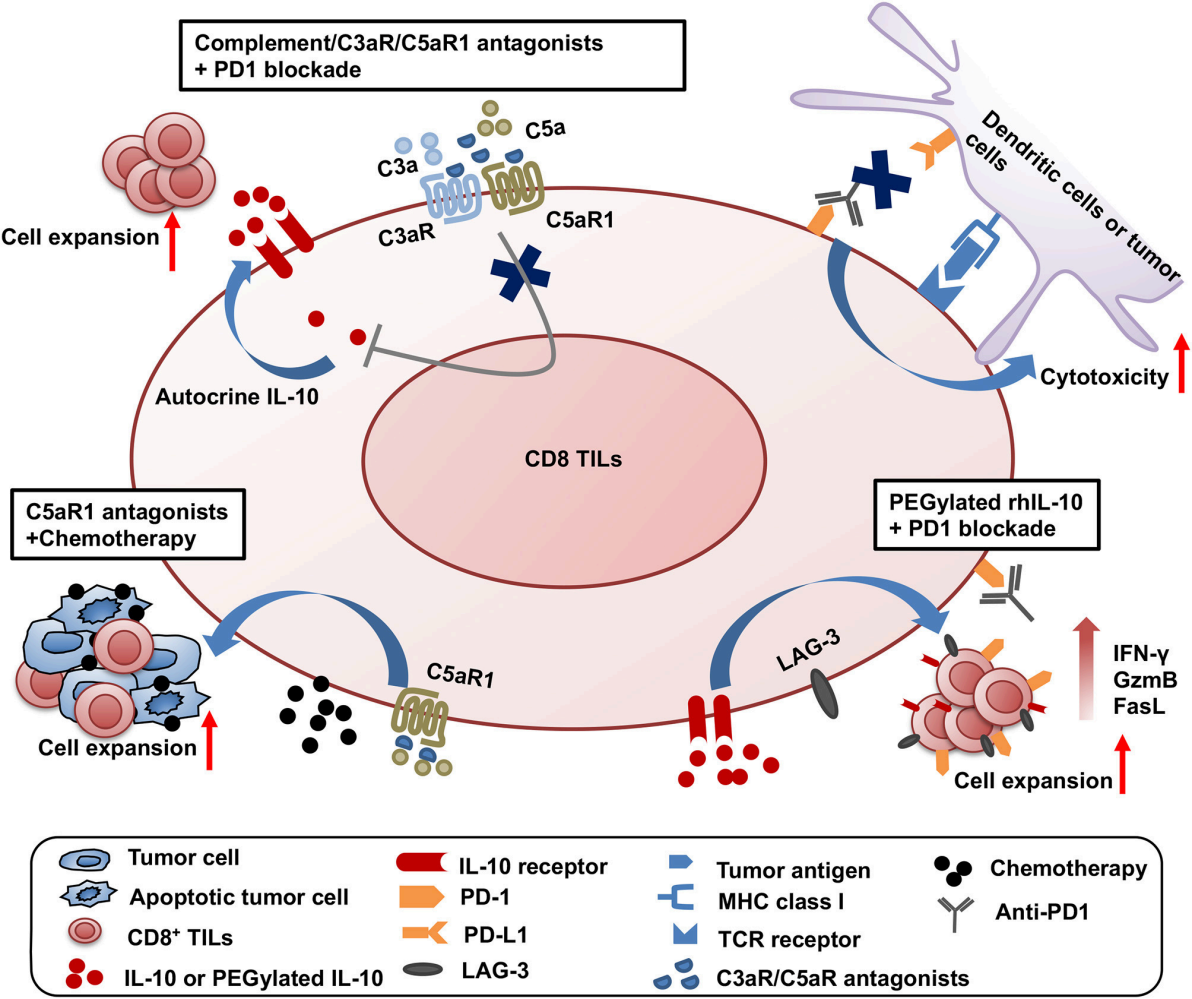
Synergistic effect of three combined strategies by targeting complement/C3aR /C5aR1/IL-10 pathway and other treatment modalities. The three combined strategies were shown as follows: (1) dual blockade of complement signaling and immune checkpoint receptor PD-1; (2) complement signaling blockade and chemotherapy; (3) the clinical use of pegylated rhIL-10 with anti-PD-1 antibody.

Significant progress has been made on testing the synergistic effect of combined treatment targeting the complement/C3aR/C5aR/IL-10 pathway and other cancer treatment modalities. The first combined strategy is dual blockade of complement signaling and immune checkpoint receptor PD-1 (Figure 1). The complement signaling/IL-10 pathway is independent of the PD-1/PD-L1 pathway as modulation of this pathway does not affect the expression of PD-1 on T cells and PD-L1 on tumor cells (11). Two different experimental systems in which (1) PD-L1-silenced B16F10 tumors were inoculated in C3-deficient mice or (2) B16F10 tumor-bearing wildtype mice were treated with anti-PD1 and antagonists to C3aR/C5aR clearly show that blockade of complement signaling and PD-1/PD-L1 interaction has dramatic synergistic antitumor effect (11). This synergistic antitumor effect is subsequently confirmed by two other studies (12, 13). These data provide important clues to rational design of future clinical trials.

The second combined treatment strategy uses complement signaling blockade and chemotherapy (Figure 1). In a squamous cell carcinoma (SCC) model, antagonist to C5aR1 enhances the treatment efficacy of paclitaxel chemotherapy and the synergistic effect depends on CD8^+^ T lymphocytes (15). Increased CD8^+^ TILs and the expansion of specific T cell clones were associated with enhanced efficacy (15).

The third combination is the clinical use of pegylated rhIL-10 with the anti-PD-1 antibody pembrolizumab in a cohort of heavily pretreated patients with melanoma, no-squamous cell lung cancer or renal cell carcinoma (61) (Figure 1). This combination achieved a 42% objective response rate, in contrast to the 21% objective response rate by pegylated rhIL-10 monotherapy (59). A combination of pegylated rhIL-10 with anti-PD-1 promotes persistent proliferation and expansion of LAG-3^+^PD-1^+^ CD8^+^ T cells in the cancer patients. These exciting clinical trial results have opened new avenues for effective cancer immunotherapy.

In summary, we have identified that tumor infiltrating CD8^+^ T cells express complement receptors C3aR and C5aR and complement signaling inhibits anti-tumor functions through repression of endogenous IL-10 production in CD8^+^ TILs. We and other groups have also confirmed that endogenous and exogenous IL-10 enhances anti-tumor functions of CD8^+^ T cells in human and mouse *in vitro* and *in vivo*. The independence of complement/C3aR/C5aR/IL-10 from the PD-1/PD-L1 signaling pathway makes it possible to block complement receptors and PD-1/PD-L1 as a combined therapy to treat cancer patients clinically. Results from other groups also suggest that the combined blockade of complement and PD-1/PD-L1 signaling with antibodies improves the efficacy of treatment through other mechanisms. Together, we and other groups provide clear evidences that complement receptors C3aR and C5aR are a new class of immune checkpoint receptors.

## Author Contributions

YW, HZ, and Y-WH co-wrote the review.

### Conflict of Interest Statement

The authors declare that the research was conducted in the absence of any commercial or financial relationships that could be construed as a potential conflict of interest.
